# Transient deoxyhemoglobin formation as a contrast for perfusion MRI studies in patients with brain tumors: a feasibility study

**DOI:** 10.3389/fphys.2024.1238533

**Published:** 2024-04-25

**Authors:** Vittorio Stumpo, Ece Su Sayin, Jacopo Bellomo, Olivia Sobczyk, Christiaan Hendrik Bas van Niftrik, Martina Sebök, Michael Weller, Luca Regli, Zsolt Kulcsár, Athina Pangalu, Andrea Bink, James Duffin, David D. Mikulis, Joseph A. Fisher, Jorn Fierstra

**Affiliations:** ^1^ Department of Neurosurgery, Clinical Neuroscience Center, University Hospital Zurich, University of Zurich, Zurich, Switzerland; ^2^ Department of Physiology, University of Toronto, Toronto, ON, Canada; ^3^ Joint Department of Medical Imaging and the Functional Neuroimaging Lab, University Health Network, Toronto, ON, Canada; ^4^ Department of Anesthesia and Pain Management, University Health Network, University of Toronto, Toronto, ON, Canada; ^5^ Department of Neurology, Clinical Neuroscience Center, University Hospital Zurich, University of Zurich, Zurich, Switzerland; ^6^ Department of Neuroradiology, Clinical Neuroscience Center, University Hospital Zurich, University of Zurich, Zurich, Switzerland

**Keywords:** MR perfusion, deoxyhemoglobin, brain tumor, glioblastoma, advanced imaging

## Abstract

**Background:** Transient hypoxia-induced deoxyhemoglobin (dOHb) has recently been shown to represent a comparable contrast to gadolinium-based contrast agents for generating resting perfusion measures in healthy subjects. Here, we investigate the feasibility of translating this non-invasive approach to patients with brain tumors.

**Methods:** A computer-controlled gas blender was used to induce transient precise isocapnic lung hypoxia and thereby transient arterial dOHb during echo-planar-imaging acquisition in a cohort of patients with different types of brain tumors (n = 9). We calculated relative cerebral blood volume (rCBV), cerebral blood flow (rCBF), and mean transit time (MTT) using a standard model-based analysis. The transient hypoxia induced-dOHb MRI perfusion maps were compared to available clinical DSC-MRI.

**Results:** Transient hypoxia induced-dOHb based maps of resting perfusion displayed perfusion patterns consistent with underlying tumor histology and showed high spatial coherence to gadolinium-based DSC MR perfusion maps.

**Conclusion:** Non-invasive transient hypoxia induced-dOHb was well-tolerated in patients with different types of brain tumors, and the generated rCBV, rCBF and MTT maps appear in good agreement with perfusion maps generated with gadolinium-based DSC MR perfusion.

## Introduction

Magnetic resonance imaging (MRI) perfusion plays an important role in the diagnostic work-up and ongoing surveillance of patients with brain tumors. Dynamic susceptibility contrast (DSC) perfusion MRI traces the passage of a bolus of contrast through the cerebral vasculature. Most commonly, the systemic injection of gadolinium-based contrast agents (GBCA) is used to acquire resting perfusion measures, such as cerebral blood volume (CBV), cerebral blood flow (CBF) and mean transit time (MTT). This can either be obtained in an absolute fashion, with the use of an arterial input function (AIF), or more commonly relatively to healthy tissue (e.g., contralateral white matter) (Boxerman et al., 2020). DSC perfusion imaging exploits the volume diffusion theory, whereby the injected bolus of contrast agent causes a transient signal drop on spin echo and gradient echo planar imaging and this can be used to infer time dependent changes in tissue concentration of contrast agent, which is relatable to tissue perfusion (Calamante, 2013). Within this theorem, the paramagnetic properties of deoxyhemoglobin (dOHb) have recently been explored as an endogenous contrast agent (Poublanc et al., 2021; Vu et al., 2021). In fact, a bolus of dOHb acts as susceptibility contrast, generating similar rCBV, rCBF, MTT perfusion maps to those obtained with GBCA in healthy subjects and in patients with cerebrovascular steno-occlusive disease (Poublanc et al., 2021; Vu et al., 2021; Sayin et al., 2022; Sayin et al., 2023a). This is obtained, as previously demonstrated, by means of a gas blender with a sequential gas delivery breathing circuit to implement rapid “bolus-like” changes in the partial pressure of oxygen of the arterial blood (Slessarev et al., 2007; Poublanc et al., 2021; Sayin et al., 2022). The viability of this technique presents potential advantages such as avoidance of an exogenous gadolinium-based contrast agent, with its connected drawbacks, in a patient population requiring serial longitudinal imaging follow-up. For this reason, after promising reports in healthy subjects and in patients with cerebrovascular steno-occlusive disease, in the present study we sought to investigate the feasibility of using transient hypoxia-induced dOHb as a contrast agent for perfusion imaging in a patient cohort with different types of brain tumors.

## Methods

The study was approved by the ethics board of the Canton of Zurich, Switzerland (research protocol KEK-ZH-No.2020-02314) and all participants provided informed consent prior to inclusion into the study. For this feasibility study, in the period from March to October 2022, subjects admitted at the Department of Neurosurgery of the University Hospital Zurich, Switzerland, with a newly diagnosed brain tumor planned for surgical resection, irrespectively of suspected tumor histology, were prospectively included to undergo a transient hypoxia induced-dOHb imaging study with a standardized breathing protocol before the intervention. At inclusion, the patients were also offered a follow-up scan. Exclusion criteria were as follows: presence of known severe cardiopulmonary disease, i.e., (severe heart insufficiency, pulmonary diffusion impairment disease, severe COPD or severe asthma), standard MRI contraindications including allergy to GBCA, pregnancy, glaucoma, metallic tattoo dyes, severe renal insufficiency and metallic prosthesis, age < 18 years, inability or refusal to sign informed consent.

### MRI protocol

Patients were scanned on a 3 T Skyra VE11 (Siemens, Erlangen, Germany) scanner with a 32-channel head coil. An axial 2D T2*-weighted gradient echoplanar sequence (50 slices with interleaved acquisition) planned on the ACPC line plus 20° anticlockwise on a sagittal image was used to acquire the BOLD data, with voxel size 2.5 × 2.5 × 2.5 mm^3^, repetition time (TR)/TE 1800/30 m, flip angle 80°, bandwidth 2168 Hz/Px, field of view (FOV) 220 × 220 mm^2^. During the echo-planar imaging (EPI) sequence acquisition, 160 volumes were acquired corresponding to a duration of circa 5 minutes. Acquisition parameters of T1-CE, FLAIR, T2 as per standard brain tumor protocol have been described in previously published article (Stumpo et al., 2021). For a subgroup of patients, clinical-diagnostic work-up required execution of a gadolinium (Dotarem) enhanced DSC-MRI GRE-EPI, FOV 100 × 100 mm^2^, resolution 128 × 128, TR/TE 2040/36, FA 90°.

### Respiratory protocol

**FIGURE 1:**
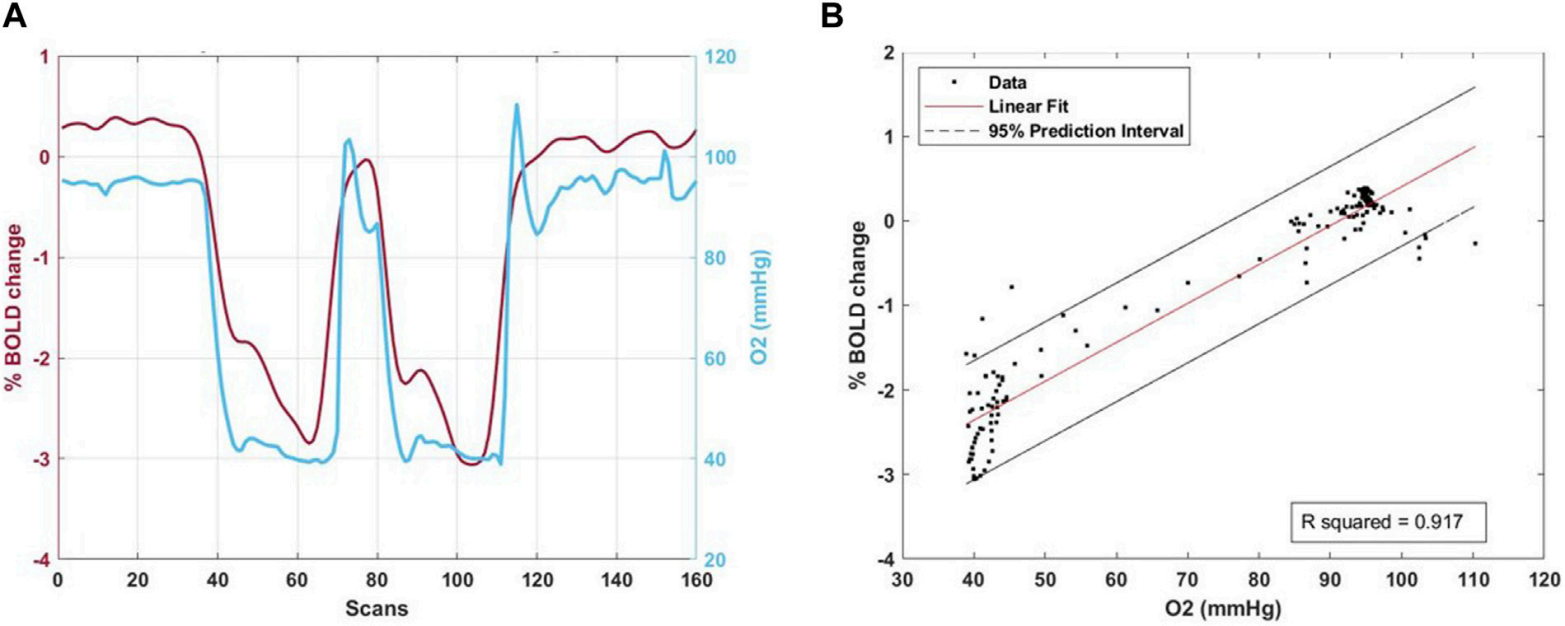
Right panel **(A)**. The percentage mean BOLD signal change in the combined grey and white matter mask plus the combined whole lesion including contrast-enhancement, necrosis and edema is shown in one patient during the hypoxic respiratory protocol. After 60 s at 95 mmHg PETO2 two hypoxic stimuli at 40 mmHg PETO2 lasting 60 s each with 20–40 s return to normoxia in between could be induced with optimal results (blue line). The corresponding percentage BOLD signal change during gas manipulation is shown (dark red line). Left Panel **(B)** shows the relative linear fit plot.

A custom-built computer controlled gas blender (RepirAct™ Gen 4, Thornhill Medical, Toronto, Canada) was used to precisely control partial pressure of end-tidal O_2_ (P_ET_O_2_) and CO_2_ (P_ET_CO_2_) with the prospective gas targeting algorithm based on (Slessarev et al., 2007). BOLD signal changes were induced by a rapid double hypoxic stimulus as previously proposed by and Sayin et al. (Poublanc et al., 2021; Sayin et al., 2022; Sayin et al., 2023a) The programmed P_ET_O_2_ stimulus pattern was 4-min and 20 s long and consisted of a 60 s baseline P_ET_O_2_ of 95 mmHg (normoxia), a step decrease in P_ET_O_2_ to 40 mmHg (hypoxia) for 60 s, a return to normoxia for 20–40 s, a second step decrease in P_ET_O_2_ to 40 mmHg for 60 s, followed by a return to normoxia for 40-60 s (see also Figure 1).

### Processing of BOLD and T1-weighted volumes

The acquired transient hypoxia induced-dOHb images were first slice-time corrected and volume re-registered for motion correction within the time-series. Next the BOLD images were realigned to their respective axial anatomical T1-weighted images using Analysis of Functional Neuroimaging (AFNI) software Version 22.0.04‘Hadrian’. (National Institutes of Health, Bethesda, Maryland) (Cox, 1996; Cox and Hyde, 1997). Using Statistical Parameter Mapping (SPM 12, Wellcome Trust Centre for Neuroimaging, Institute of Neurology, University College London; http://www.fil.ion.ucl.ac.uk/spm), automated segmentation of the T1-weighted image yielded grey and white matter, cerebrospinal fluid, skull and skin probability maps. T2, FLAIR, and T1-CE volumes were co-registered and resliced to the high-resolution T1 image acquired in the same session as the BOLD.

### Calculation of resting perfusion maps

For quality control, we calculated the goodness of fit (Rsquared) of the percentage BOLD signal change and the P_ET_O_2_ time series was regressed using a linear least square fitting to the BOLD time series on a voxel-per-voxel basis.

Perfusion measures using transient hypoxia induced-dOHb were calculated with a conventional analysis using an arterial input function (AIF) chosen over the middle cerebral artery and a deconvolution-based model as previously described by (Poublanc et al., 2021). First, an AIF was chosen over a voxel in the middle cerebral artery. The signal was smoothed voxel-wise using an adaptive mean filtering of width 7 mm. The tracer kinetics relationship was applied in the signal domain and rCBV and MTT metrics were determined using a least square fitting procedure, with MTT bound between 1 and 8 s. The model uses a mono-exponential residue function. Using the central volume theorem, rCBF was then calculated as rCBF = rCBV/MTT (Østergaard, 2005) and scaled by 30 arbitrary units (a.u.).

### Automatic segmentation and region-of-interest (ROI) determination

Region of interest masks (contrast enhancement, necrosis and edema) were automatically segmented using Oncohabitats Software (Juan-Albarracín et al., 2018) for glioblastomas and manually segmented for the other tumor types. Automatically segmented tumor region-of-interest were visually inspected for accuracy and required no subsequent manual correction.

### Calculation of perfusion measures in ROIs

MRI volumes were analyzed using MATLAB 2019 (The MathWorks, Inc., Natick, United States). rCBV, rCBF and MTT values were calculated in the grey and white matter after masking out the whole tumor (including edema) and for each of the aforementioned tumor ROIs. Two additional ROI were defined: tumor, i.e., contrast-enhancement + necrosis and whole lesion, including surrounding edema; and the corresponding volumes in the contralateral hemisphere identified using proprietary Matlab programme. Perfusion metrics were overlayed onto their respective anatomical images using SPM software and qualitatively compared to the co-registered T1CE and FLAIR to identify perfusion patterns in tumor ROIs (contrast enhancement, edema, necrosis).

### DSC-MRI analysis

In a subgroup of patients, a DSC-MRI was acquired during clinical workup for diagnostic purposes. The acquired sequences were analyzed (including leakage correction) using Olea Software version 3.0 to calculate rCBV, rCBF, MTT maps. The perfusion maps were exported, co-registered and resliced to the high-resolution T1 anatomical images and qualitatively compared with the resting perfusion measures obtained with transient hypoxia induced-dOHb. Average rCBV, rCBF and MTT were calculated in the same above-mentioned ROI and contralateral flipped masks and compared with transient hypoxia induced-dOHb estimated values.

## Results

### Study population

Eleven patients provided informed consent for participation in the study. Of these, nine patients completed the imaging protocol and were included in the final study population, reasons of dropout of the two excluded patients being in one patient interruption of study due to dyspnea during the respiratory protocol, in the other discomfort and claustrophobia during MRI acquisition. One patient underwent the imaging protocol twice, the second being after tumor resection. Mean age was 56 (SD 13.8; 3 female subjects). The baseline characteristics are provided in Table 1.

**TABLE 1:** Study population.

Identifier	Age	Sex	Tumor	Side	Location	Tumor volume (mL)	Edema volume (mL)	Gad-DSC
**1**	**77**	**M**	**Lung adenocarcinoma metastasis**	**L**	**Frontal**	**11.1**	**10.7**	**No**
**2**	**58**	**M**	**Glioblastoma**	**L**	**Frontobasal**	**41.3**	**25.9**	**Yes**
**3**	**59**	**M**	**Astrocytoma PXA G3**	**R**	**Mediobasal**	**64.0**	**49.1**	**No**
**4a**	**60**	**F**	**Glioblastoma**	**L**	**Multifocal (precuneus, cuneus)**	**17.9**	**10.2**	**Yes**
**4b**	**-**	**-**	**-**	**-**	**-**	**-**	**-**	**Yes**
**5**	**51**	**F**	**Oligodendroglioma G3**	**R**	**Frontobasal**	**82.6**	**50.1**	**No**
**6**	**57**	**M**	**Glioblastoma**	**L**	**Superior temporal gyrus**	**18.6**	**12.8**	**No**
**7**	**29**	**F**	**Astrocytoma G2**	**R**	**Middle temporal gyrus**			**Yes**
**8**	**41**	**M**	**Meningioma**	**L**	**Falx**	**42.7**	**41.9**	**No**
**9**	**72**	**M**	**Glioblastoma**	**L**	**Temporopolar**	**63.2**	**47.0**	**Yes**

Abbreviations. F, female; G, grade; Gad, gadolinium; L, left; M, male; mL, milliliter; PXA, pleomorphic xanthoastrocytoma; R, right; DSC, dynamic susceptibility contrast.

The study population included patients with different types of brain tumors: 1 metastasis, 1 meningioma, 1 astrocytoma WHO grade 2, 1 pleomorphic xanthoastrocytoma WHO grade 3, 1 oligodendroglioma WHO grade 3, 4 glioblastomas (1 patient was also scanned post-operatively). The histopathological diagnosis was based on the 2021 WHO classification (Louis et al., 2021). Patient 3 was excluded from the following analysis due to excessively noisy BOLD signal, caused by suboptimal stimulus during the examination. Four of the included patients received during diagnostic work-up a standard gadolinium perfusion MRI, with one of them receiving it at another center before referral.

An illustrative BOLD signal change and linear fit plot is shown in Figure 1 and is reported for each patient in Supplementary Figure S1.

### Resting transient hypoxia induced-dOHb resting perfusion measures

Transient hypoxia induced-dOHb resting perfusion measures in the abovementioned regions-of-interest were calculated for each patient as previously reported (Poublanc et al., 2021). The relative measurements are reported in Table 2.

**TABLE 2:** Transient hypoxia induced-dOHb perfusion measures in Gray Matter, White Matter, Contrast-Enhancement, Flipped Contrast-Enhancement, Edema, Flipped Edema of included patients.

	GM			WM			CE			Flipped CE			Edema			Flipped edema		
	MTT	CBV	CBF	MTT	CBV	CBF	MTT	CBV	CBF	MTT	CBV	CBF	MTT	CBV	CBF	MTT	CBV	CBF
Identifier																		
**1**	**2.7**	**2.0**	**45.6**	**3.2**	**1.8**	**35.0**	**3.6**	**2.4**	**67.3**	**5.4**	**1.0**	**15.7**	**5.9**	**1.6**	**24.0**	**6.9**	**1.3**	**13.5**
**2**	**3.7**	**4.4**	**59.8**	**3.9**	**4.3**	**59.2**	**3.8**	**5.2**	**71.2**	**3.8**	**3.2**	**55.1**	**2.6**	**4.0**	**69.9**	**3.6**	**3.7**	**74.0**
**4a**	**4.2**	**5.2**	**61.6**	**4.9**	**4.1**	**46.8**	**4.8**	**7.8**	**94.1**	**6.5**	**4.3**	**42.2**	**4.7**	**4.6**	**46.6**	**5.4**	**4.3**	**42.5**
**5**	**3.8**	**4.3**	**55.5**	**4.4**	**3.0**	**39.3**	**5.0**	**12.7**	**122.6**	**5.2**	**6.4**	**63.7**	**4.8**	**9.0**	**93.3**	**5.1**	**10.5**	**108.4**
**6**	**5.6**	**1.7**	**16.3**	**6.2**	**1.3**	**14.1**	**5.2**	**4.9**	**49.2**	**5.9**	**3.5**	**29.8**	**5.7**	**1.7**	**14.8**	**5.9**	**1.8**	**17.3**
7*	**3.1**	**2.6**	**46.2**	**3.9**	**2.0**	**34.4**	**-**	**-**	**-**	**-**	**-**	**-**	**-**	**-**	**-**	**-**	**-**	**-**
**8**	**1.9**	**2.2**	**95.2**	**2.0**	**2.0**	**83.1**	**1.6**	**4.5**	**210.2**	**2.3**	**1.6**	**61.9**	**1.4**	**2.6**	**132.9**	**1.3**	**1.4**	**74.4**
**9**	**2.7**	**1.1**	**30.2**	**2.9**	**1.0**	**29.2**	**3.4**	**1.4**	**34.6**	**3.8**	**0.9**	**23.3**	**2.6**	**1.1**	**32.6**	**2.9**	**1.0**	**32.1**
**Mean (SD)**	**3.5 (1.1)**	**2.9 (1.4)**	**51.3 (21.9)**	**3.9 (1.2)**	**2.4 (1.2)**	**42.6 (19.6)**	**4.0 (1.2)**	**5.3 (3.6)**	**90.7 (56.7)**	**4.6 (1.3)**	**2.7 (1.9)**	**39.5 (20.0)**	**3.7 (1.5)**	**3.6 (2.5)**	**63.8 (35.5)**	**4.1 (4.1)**	**3.5 (3.1)**	**57.3 (29.2)**
**Min-Max**	**1.9–3.8**	**1.1–5.2**	**16.3–95.2**	**2.0–6.2**	**1.0–4.3**	**14.1–83.1**	**1.6–5.2**	**1.4–12.7**	**34.6–210.2**	**2.3–6.5**	**0.9–6.4**	**15.7–63.7**	**1.4–5.9**	**1.1–4.6**	**14.8–93.3**	**1.3–6.9**	**1.0–10.5**	**13.5–108.4**

Abbreviations. CBF, cerebral blood flow; CBV, cerebral blood volume; Max, maximum; Min, minimum; MTT, mean transit time; SD, standard deviation.

### Qualitative visual analysis

**FIGURE 2:**
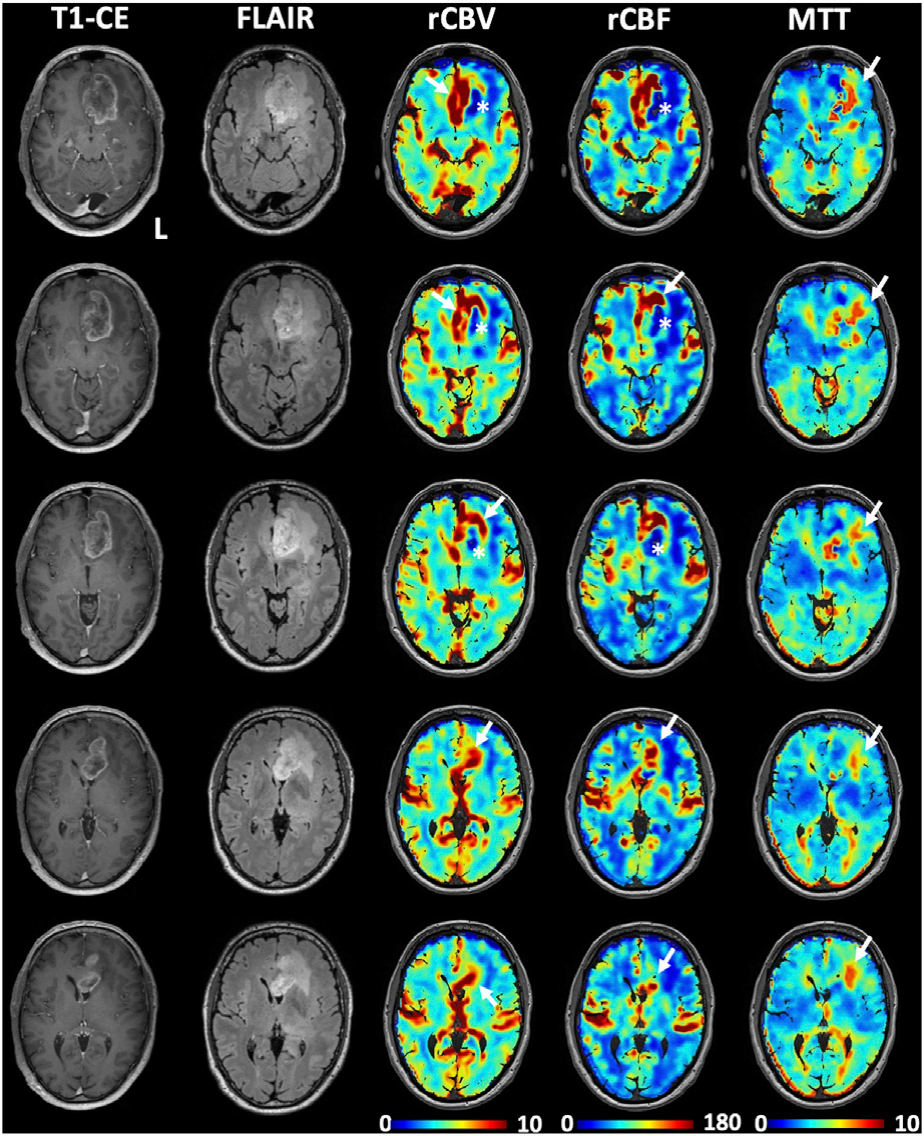
Illustrative perfusion maps obtained with the transient hypoxia induced-dOHb protocol during BOLD-MRI acquisition in a patient with left frontal glioblastoma. High perfusion, as represented by increased CBV and CBF, can be observed in correspondence of the contrast-enhancing lesion (white arrows), while decreased rCBV and rCBF can be observed in the central necrotic area (white asterisks) as well as the in the FLAIR hyperintensity typical of perilesional edema. Note that only a partial correspondence of increased perfusion is observed at the posterolateral margin of the contrast-enhancing lesion. High MTT values can be observed mostly in the perilesional edema (white arrows in the MTT maps).

**FIGURE 3:**
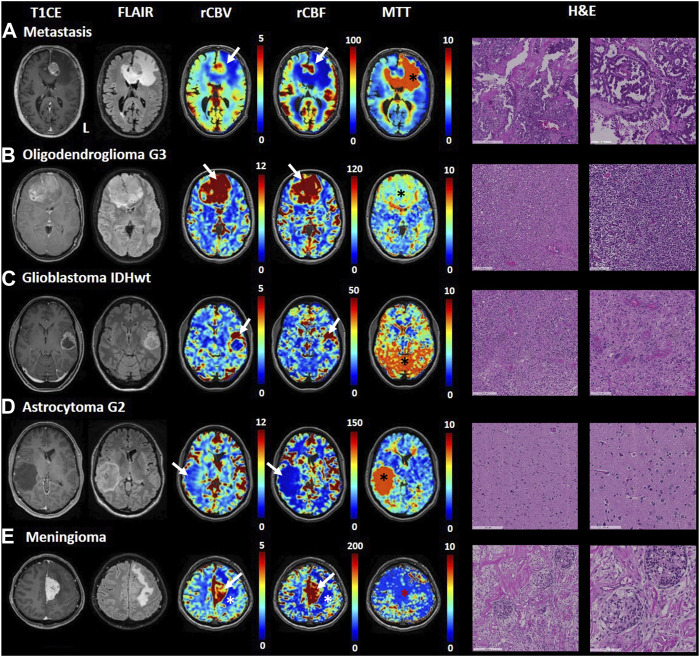
Transient hypoxia induced-dOHb MRI perfusion patterns in some common brain tumors. T1-CE, T2-FLAIR, rCBV, rCBF and MTT transient hypoxia induced-dOHb MRI perfusion maps and H&E sections are displayed for each tumor type included. **(A)**. Patient with a left frontal lung adenocarcinoma metastasis. Around the contrast-enhancing lesion, displaying high CBV and CBF (white arrow) can be appreciated an extensive perilesional edema (FLAIR hyperintensity, low CBV and CBF, high MTT - black asterisk). **(B)**. Patient with frontobasal oligodendroglioma grade 3, displaying strongly increased CBV and CBF (white arrows) as well as moderately increased MTT (black asterisk). **(C)**. Patient with left superior temporal gyrus glioblastoma. The contrast-enhancing lesion (high CBV and CBF, white arrows) surrounding a central necrotic area can be appreciated. The MTT map shows spot-like increased values, particularly evident in the posterior circulation (black asterisk) which we speculate could be attributed to noisier signal/suboptimal stimulus, **(D)**. Patient with right middle temporal gyrus astrocytoma grade 2. The lesion does not show contrast uptake, is hyperintense in FLAIR sequence and displays lower perfusion than healthy parenchyma (low CBV and CBF shown by white arrow) as well as strongly increased mean transit time (black asterisk). **(E)**. Patient with a left-sided falx meningioma. FLAIR sequence shows hyperintense edema around the contrast-enhancing lesion (note increased CBV and CBF as pointed by white arrows). The MTT map shows no increased mean transit time in the lesion (red asterisk).

Figure 2 shows an illustrative case, i.e., patient 2, 58 y. o. male with a left frontobasal glioblastoma. The perfusion maps show high CBV, CBF in the contrast-enhanced lesion regions, while these metrics are very low in the central necrotic area with high agreement with the morphological high-resolution sequences. MTT is mostly increased in peritumoral edematous tissue. Figure 3 displays perfusion patterns in different tumor types. Qualitative image analysis shows perfusion to be higher in grey matter than in white matter and increased CBV and CBF were observed in contrast-enhancing lesion of metastases (Figure 3A), oligodendroglioma WHO grade 3 (Figure 3B), glioblastoma (Figure 3C) and meningioma (Figure 3E) with respect to healthy tissue. On the contrary, lower CBV and CBF were observed in the astrocytoma WHO grade 2 (Figure 3D). MTT was shown to be higher in areas of edema identified by T2 FLAIR (Figures 3A, B) as well as in the astrocytoma WHO grade 2 dense cellular tissue (Figure 3D).

### Comparison of transient hypoxia induced-dOHb BOLD and DSC-MRI

**FIGURE 4:**
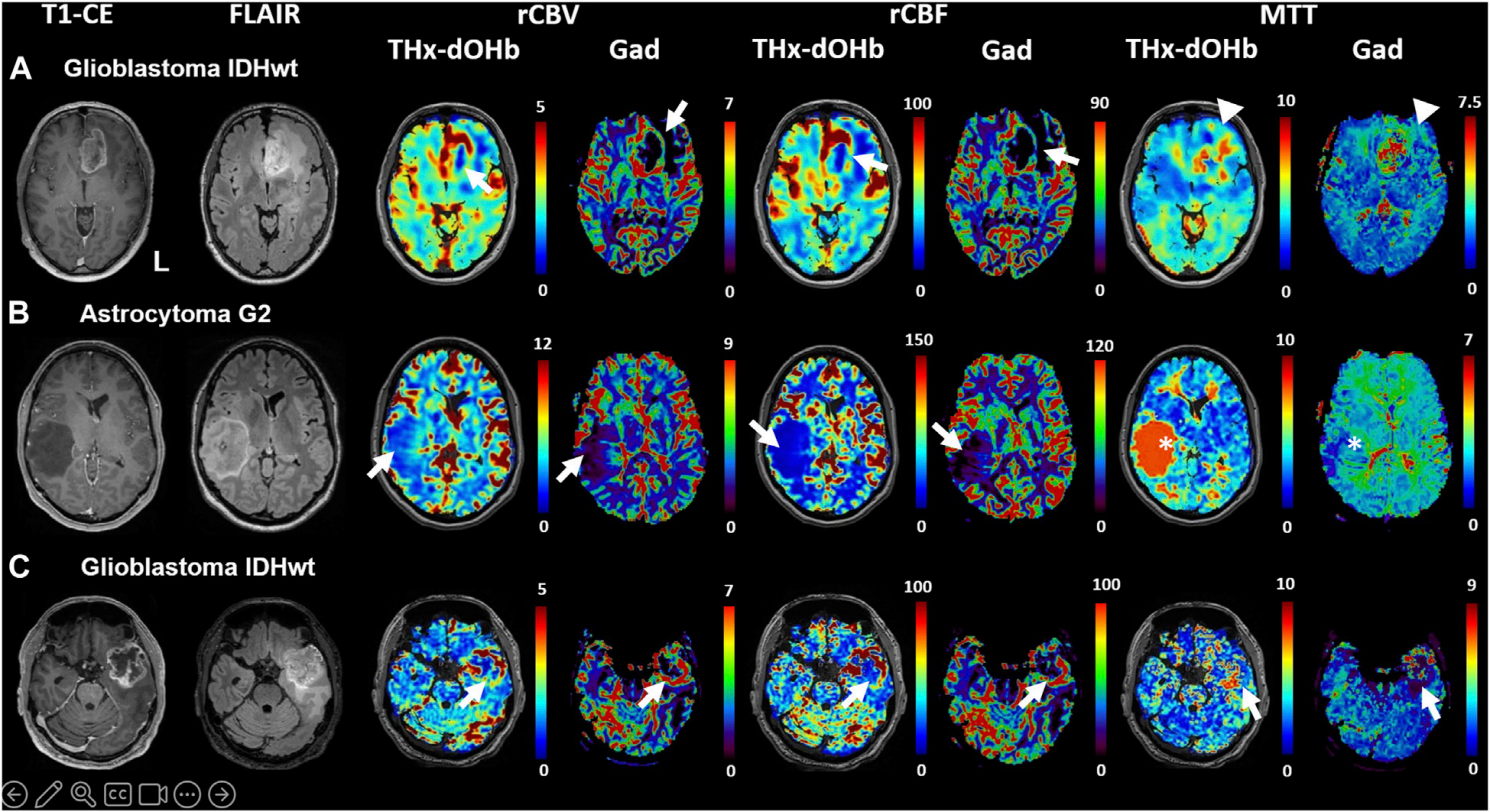
Comparison of transient hypoxia induced-dOHb perfusion maps with DSC-Gad MRI perfusion maps in three illustrative patients with diffuse cerebral glioma. T1-CE, T2-FLAIR, rCBV, rCBF and MTT obtained with transient hypoxia induced-dOHb and standard gadolinium MRI perfusion maps are displayed for three patients with cerebral diffuse glioma. **(A)**. Patient 2, left frontobasal glioblastoma. **(B)**. Patient 7, right middle temporal gyrus astrocytoma G2. **(C)**. Patient 9, left temporopolar glioblastoma. Arrowheads pointing at relevant tumor areas to facilitate comparison between transient hypoxia induced-dOHb and gadolinium perfusion. Note: There is excellent congruence between Gd and d-OHb maps except for patient B, where tumor MTT values are much higher in the transient hypoxia induced-dOHb perfusion maps as compared to the gadolinium perfusion (asterisk). The reason for this is uncertain but the finding could point to higher sensitivity of the dOHb method to MTT abnormalities.

**FIGURE 5:**
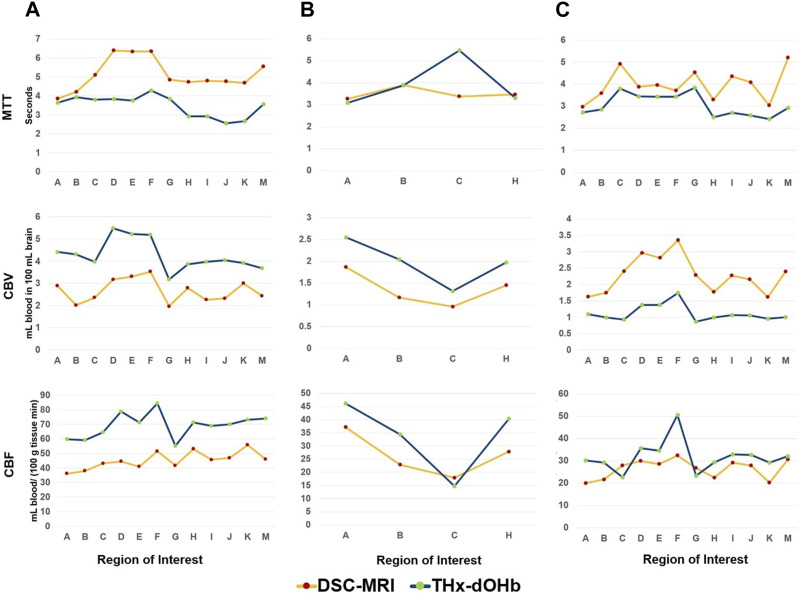
Comparison of transient hypoxia induced-dOHb perfusion and DSC-MRI measurements in selected region-of-interest in 3 patients with brain tumor. The measurements of rCBV, rCBF and MTT in grey and white matter, selected tumor ROI and contralateral flipped masks are shown for patients 2 **(A)**, 7 **(B)** and 9 **(C)** for both transient hypoxia induced-dOHb and DSC-MRI. Note that for Patient 7, i.e., middle temporal gyrus astrocytoma grade 2, only four ROIs are shown as in this tumor type no contrast-enhancement, necrosis or perilesional edema could be identified consistent with the radiological presentation of this tumor entity. Legend. **(A)**. Grey Matter, **(B)**. White Matter, **(C)**. Whole Lesion (Contrast-Enhancement + Necrosis + Edema), **(D)**. Tumor Lesion (Contrast-Enhancement + Necrosis), **(E)**. Contrast-Enhancement, **(F)**. Necrosis, **(G)**. Edema, **(H)**. Contralateral Whole Lesion, **(I)**. Contralateral Tumor, **(J)**. Contralateral Contrast-Enhancement, **(K)**, Contralateral Necrosis, **(M)**. Contralateral Edema.

In the three patients who underwent a pre-operative DSC-MRI in our institution, this was compared with the transient hypoxia induced-dOHb perfusion maps for visual qualitative examination and rCBV, rCBF and MTT were calculated in each of the two techniques. The transient hypoxia induced-dOHb BOLD perfusion and corresponding gadolinium DSC-MRI are shown for three patients (2 glioblastomas and 1 astrocytoma WHO grade 2) in Figure 4. The values of rCBV, rCBF, MTT obtained by gadolinium-based DSC-MRI and analyzed with Olea-Software were compared to the transient hypoxia induced-dOHb estimated values in grey matter, white matter, identified tumor ROIs and flipped masks (Figure 5). In particular, the plots show congruent measurements in the different ROIs and contralateral flipped masks between the two techniques with similar trends.

**FIGURE 6:**
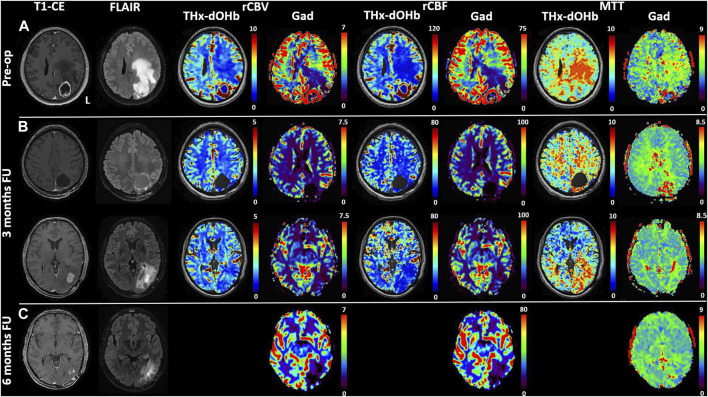
Comparison of transient hypoxia induced-dOHb and DSC-Gad MRI perfusion maps in a glioblastoma patient before resection **(A)** and during follow-up at 3 **(B)** and 6 months **(C)**. Baseline and follow-up comparison of transient hypoxia induced-dOHb and gadolinium DSC-MRI perfusion maps in patient with multifocal glioblastoma with lesions in left cuneus and precuneus. Pre-operative T1-CE, FLAIR and transient hypoxia induced-dOHb perfusion as well as corresponding gadolinium DSC-MRI maps **(A)** as well as 3 months **(B)** and 6 months follow-up MRI **(C)**, only DSC-MRI available). The patient was referred to our center after receiving an MRI on prescription from the general practitioner following persistent parietal headaches episodes, vertigo, visual disturbances, memory loss and speech difficulties. The first MRI showed, as depicted in Figure 5, a contrast-enhancing lesion surrounding a central necrotic area in the left precuneus with extensive perilesional edema as well as a smaller contrast-enhancing lesion in the left cuneus. Consistently with known higher perfusion in glioblastomas, both DSC-MRI and transient hypoxia induced-dOHb showed higher rCBV and rCBF in tumor tissue. Gross total resection of the two lesions was performed. Histological analysis confirmed the diagnosis of glioblastoma. As a consequence, after surgery the patient received concomitant radiochemoterapy with temozolomide followed by maintenance chemotherapy as per standard of care (Stupp Protocol). MGMT promoter methylation analysis revealed an unmethylated promoter. At 3 months follow-up, both DSC and transient hypoxia induced-dOHb MRI were repeated. Figure 5 (upper panel) shows the resection cavity with normal perfusion at its margins. The same follow-up MRI showed however an area of contrast enhancement surrounded by FLAIR hyperintensity caudally to the resected tumor. This lesion did not display neither in DSC- nor transient hypoxia induced-dOHb MRI increased perfusion, with the two techniques showing perfusion maps with good qualitative agreement. (Figure 5, lower panel). Another MRI performed at 6 months showed a regression of contrast enhancement and edema. This clinical case shows how transient hypoxia induced-dOHb preliminarily achieved good agreement with gadolinium DSC perfusion also in the follow-up of a glioblastoma patient, warranting further longitudinal validation in the follow-up of treated glioma patients as well as in treated brain metastases.

Based on the availability of 2 transient hypoxia induced-dOHb perfusion scans for patient 4, a multifocal glioblastoma with lesions in left cuneus and precuneus, follow-up imaging from pre-to post-operative is shown and compared with gadolinium DSC (Figure 6). Transient hypoxia induced-dOHb BOLD perfusion maps display good spatial agreement with gadolinium-based DSC perfusion also in the longitudinal follow-up of a glioblastoma patient.

## Discussion

### Summary of study findings

Our preliminary study shows good feasibility of transient controlled hemoglobin desaturation by means of a standardized hypoxic stimulus to obtain resting perfusion parameters in a population of patients with brain tumor. At the single patient level, we found the calculated transient hypoxia induced-dOHb perfusion maps to be in good agreement with intrinsic tumor characteristics that would be expected in a GBCA perfusion, i.e., increased CBV and CBF in highly perfused tumors such as brain metastasis, glioblastomas, oligodendroglioma and meningioma compared to healthy tissue; decreased CBV, CBF in astrocytoma grade 2 as well as increased MTT in edematous or dense cellular tissue. For the subgroup of patients for whom a DSC-MRI was available, the relative ROI-level patterns estimated using transient hypoxia induced-dOHb well reflect the measurement obtained in standard gadolinium perfusion. Moreover, the longitudinal assessment in one patient shows good qualitative agreement with the relative DSC-MRI. The investigated technique, which exploits the BOLD-related drop in MRI signal induced by a bolus of paramagnetic deoxyhemoglobin as a possible alternative for gadolinium contrast, has been recently introduced in seminal publications by (Vu et al., 2021; Poublanc et al., 2021; Sayin et al., 2022) in healthy subjects as well as in patients with steno-occlusive cerebrovascular disease (Sayin et al., 2023a).

### Perfusion MRI in brain tumor assessment–“status quo”

Perfusion assessment is clinically relevant in brain tumor diagnosis and follow-up, as increased perfusion correlates with tumor’s aggressiveness and can be used in the differential diagnosis of cerebral lesions as well as to distinguish post-treatment changes from tumor recurrence/progression (Guida et al., 2022; Stumpo et al., 2022). However, in a proportion of brain tumors the blood brain barrier is either disrupted or dysfunctional (e.g., glioblastomas, metastasis) (Arvanitis et al., 2020; Guan et al., 2021), making contrast leakage in the extravascular extracellular space a significant confounder to reliable and reproducible measurements (Shiroishi et al., 2015; Leu et al., 2017; Boxerman et al., 2020). For this reason, GBCA perfusion techniques require appropriate correction and rely most commonly on a loading dose of contrast agent administered prior to the acquisition to minimize T1 changes during first pass, and/or on mathematic correction algorithms (Leu et al., 2017; Boxerman et al., 2020).

### Transient hypoxia induced-dOHb for perfusion assessment: differences from gadolinium perfusion–technical aspects, model and potential

Previous literature has focused on BOLD-contrast for functional MRI and task-based pre-surgical mapping (as neural activation induces changes in regional blood flow) (Glover, 2011; Zacà et al., 2014), or resting-state fMRI for functional architecture inference (Lee et al., 2013; Stoecklein et al., 2020), as well as characterization of cerebrovascular reactivity during vasodilatory stimulus (Fierstra et al., 2013; Fierstra et al., 2016; Fierstra et al., 2018; Fisher et al., 2018; Muscas et al., 2020; Sebök et al., 2020; Sebök et al., 2021). Only recently has the possibility to induce a reproducible and standardized transient bolus of deoxygenated blood (while controlling for competing PCO_2_ effects) - in combination with the high temporal resolution of EPI sequence - become focus of attention in the setting of perfusion quantification (Poublanc et al., 2021; Vu et al., 2021; Sayin et al., 2022; Sebök et al., 2023a). BOLD imaging contrast is well suited for this technique as it derives from the different physical properties of hemoglobin in its saturated and desaturated state which result in a diamagnetic *versus* a paramagnetic signal response, respectively (Buxton, 2013; Gauthier and Fan, 2019). Perfusion patterns observed in our study are in agreement to what is reported in the literature for different tumor histologies (Tamrazi et al., 2016; Guida et al., 2022; Stumpo et al., 2022) and display, even if with some spatial differences, remarkably good qualitative agreement with DSC-MRI. To which extent the spatial as well as quantitative differences observed are determined by intrinsic tumor characteristics exhibiting differential response to a contrast medium *versus* an endogenous deoxyhemoglobin bolus remains to be further investigated in future studies. Nevertheless, development and validation of the transient hypoxia induced-dOHb technique for perfusion measurement in patients with brain tumors would present several benefits with respect to gadolinium contrast. We expect that endogenous deoxyhemoglobin, as opposed to GBCA, remains completely intravascular even in cases of BBB leakage. As such it could be even more sensitive to certain vascular properties of some tumors with respect to gadolinium, despite caution need to be taken due to the described magnetic distortion generated by deoxyhemoglobin also in the extravascular space. (Buxton, 2013). The potential advantages of this technique include avoidance of an exogenous contrast agent with its connected drawbacks, i.e., potential of allergic reactions (Gracia Bara et al., 2022), known accumulation of gadolinium in the brain (Gulani et al., 2017), difficult handling in nephropathic patients due to concerns of nephrotoxicity (Weinreb et al., 2021) and a higher repeatability of the scan during treatment follow-up. These benefits are also shared from arterial spin labeling (ASL) MRI, which in recent years has been extensively investigated in the setting of cerebral perfusion assessment and also in patients with brain tumor. Despite promising results, this technique lagged somewhat behind in the clinical implementation. (Cebeci et al., 2014; Alsop et al., 2015; Haller et al., 2016; Alsaedi et al., 2018).

On the other hand, possible drawbacks include susceptibility artifacts that limit the study of tumors with hemorrhagic component (e.g., melanoma metastasis), reduced contrast to noise ratio and higher predisposition to movement artifacts. Optimization of pre-processing steps (smoothening, etc.) should be also pursued to find the optimal trade-off between decreasing noise and maintaining a fair spatial specificity useful in brain tumor assessment. In order to characterize the relative effect of such confounders, as well as differences and similarities between traditional DSC-MRI *versus* transient hypoxia induced-dOHb, future validation studies should ideally adopt the same acquisition parameters.

Further, blood desaturation for investigation of brain tumor vascularity could exploit analysis technique different than bolus tracking method such as carpet plot analysis (Bhogal et al., 2022) or transfer function analysis (Sayin et al., 2023b), with these having already provided encouraging results in healthy subjects. Moving away from resting perfusion assessment, other approaches may be employed to investigate intrinsic tissue features by dissecting specific BOLD signal changes characteristics during gas control. A preliminary study demonstrated the feasibility of such technique, with a small cohort of glioblastoma patients exhibiting unique tissue response patterns during hypoxic, hyperoxic and hypercapnic stimuli (Stumpo et al., 2021). For this reason, a longitudinal cohort study after optimization of the hypoxic stimulus is currently ongoing. The potential to better characterize tumoral tissue and peritumoral tissue infiltration with such approach relies not only on the observed magnitude of signal change during evoked stimulus, but also the complementary information derived from a refined analysis including other parameters such as goodness-of-fit, contrast-to-noise ratio and temporal lag. In fact, these variables allow further categorization of dynamic functional tissue characteristics that may not be appreciated in static conditions by traditional perfusion analysis. Moreover, advanced data-driven analysis methods such as time-series clustering could also be exploited to identify subgroups of voxels based on stimulus-evoked hemodynamic patterns to correlate with physiological *versus* pathological vasculature and underlying histological and functional properties of the tissue studied (Baudelet and Gallez, 2003).

## Limitations

Our study has several limitations. Despite visual analysis of the calculated maps show high similarity with expected perfusion patterns in different tumors, its qualitative nature as well as the small sample size prevents us from drawing more robust conclusions on the underlying quantitative data. Moreover, at the present stage, while not requiring the injection of a contrast agent, this technique has other drawbacks including longer post-processing times as compared with the clinically available software for DSC-MRI perfusion analysis, higher costs related to gas and masks procurement and, lastly, potential reduced tolerance to the mask and to the hypoxic stimulus may also lead to higher dropout rate in poorly cooperative patients. The time required for the acquisition of the EPI sequence is not significantly longer than the one used in clinical setting for the DSC perfusion. Of note, extensive literature in DSC-gadolinium perfusion MRI described thoroughly confounders of absolute perfusion quantification in brain tumors due to a variety of factors (e.g., flip angle, echo time, temporal resolution, baseline and post-bolus data points, post-processing techniques and leakage correction, etc.) (Boxerman et al., 2020). In this context, the results of our preliminary qualitative analysis warrant future both intra- and inter-subject repeatability assessment. Only 4 patients included in our study received a gadolinium-contrast DSC-MRI to compare transient hypoxia induced-dOHb perfusion and with different acquisition parameters, with one of them receiving it at an external institution. Regardless, qualitative analysis in these 4 patients suggests high concordance of gadolinium perfusion and deoxyhemoglobin-based perfusion with inter-ROI relative measurement showing similar patterns between the two techniques. A validation study in a larger patient population with defined tumor subgroups is currently underway to validate the quantitative measurements obtained by deoxyhemoglobin perfusion against a clinical DSC-MRI gadolinium perfusion.

## Conclusion

In this feasibility study, transient and precise hemoglobin desaturation by controlled hypoxic gas modulation is feasible and repeatable in patients with brain tumor. The induced signal change allows for resting brain tissue perfusion measurements, which qualitatively are in good agreement with gadolinium-based perfusion in the study cohort. Based on promising preliminary data, deoxyhemoglobin-based perfusion warrants further quantitative validation against gadolinium in a larger, heterogenous cohort of patients with brain tumor.

## Data Availability

The raw data supporting the conclusions of this article will be made available by the authors upon reasonable request.
