# Supplementary figures and images for: Transient deoxyhemoglobin formation as a contrast for perfusion MRI studies in patients with brain tumors: a feasibility study
Source: Front Physiol. 2024 Apr 25;15:1238533. doi: 10.3389/fphys.2024.1238533 (PMC11079274; doi:10.3389/fphys.2024.1238533)

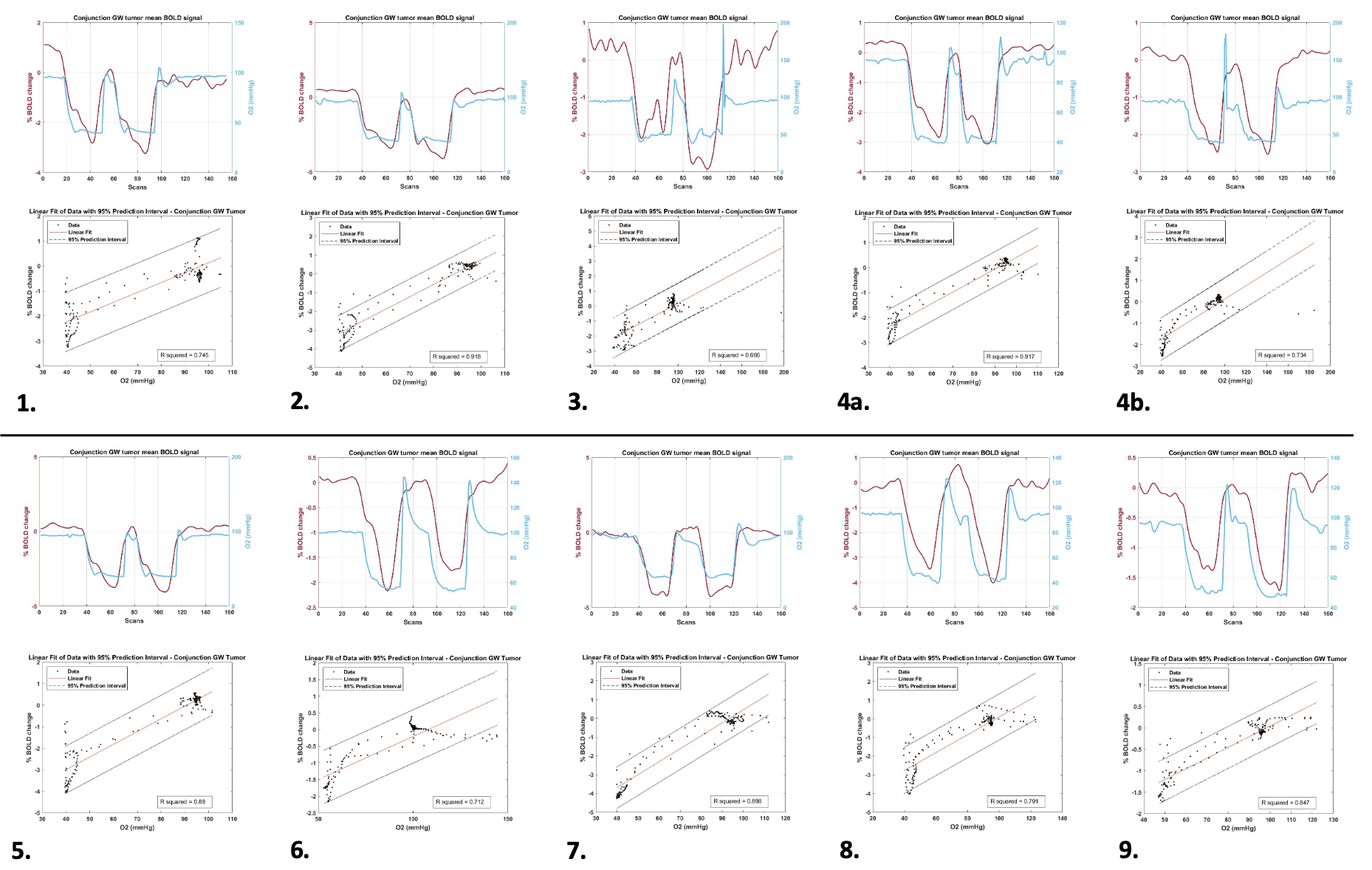

Supplement: Supplementary file 1 [file Image1.tiff]
